# Prediction models combining zonulin, LPS, and LBP predict acute kidney injury and hepatorenal syndrome–acute kidney injury in cirrhotic patients

**DOI:** 10.1038/s41598-023-40088-7

**Published:** 2023-08-11

**Authors:** Yi-Hsuan Lin, Nai-Rong Kuo, Hsiao-Chin Shen, Yun-Chien Chang, Roger Lin, Tsai-Ling Liao, Hsiao-Yun Yeh, Ying-Ying Yang, Ming-Chih Hou, Han-Chieh Lin

**Affiliations:** 1 Department of Medical Education, Medical Innovation and Research Office, Clinical Innovation Center, Taipei Veterans General Hospital, Taipei, Taiwan; 2 Faculty of Medicine, School of Medicine, National Yang Ming Chiao Tung University, Taipei, Taiwan; 3 Department of Family Medicine, Taipei Veterans General Hospital, Taipei, Taiwan; 4 Department of Medical Research, Taichung Veterans General Hospital, Taichung, Taiwan; 5 Department of Medicine, Taipei Veterans General Hospital, Taipei, Taiwan; 6 Institute of Clinical Medicine, National Yang Ming Chiao Tung University, Taipei, Taiwan

**Keywords:** Gastroenterology, Nephrology

## Abstract

The development of acute kidney injury (AKI) and hepatorenal syndrome–acute kidney injury (HRS–AKI) in cirrhosis has been associated with intestinal barrier dysfunction and gut-kidney crosstalk. We use the related markers such as zonulin, lipopolysaccharides (LPS), and lipopolysaccharide-binding protein (LBP) to predict AKI and HRS–AKI in cirrhotic patients and evaluate their in vitro effects on intestinal (Caco-2) cells and renal tubular (HK-2) cells. From 2013 to 2020, we enrolled 70 cirrhotic patients and developed prediction models for AKI and HRS–AKI over a six-month period. There were 13 (18.6%) and 8 (11.4%) cirrhotic patients developed AKI and HRS–AKI. The prediction models incorporated zonulin, LPS, LBP, C-reactive protein, age, and history of hepatitis B for AKI, and zonulin, LPS, LBP, total bilirubin, and Child–Pugh score for HRS–AKI. The area under curve (AUC) for the prediction of AKI and HRS–AKI was 0.94 and 0.95, respectively. Furthermore, the conditioned medium of LPS+hrLBP pre-treated Caco-2 cells induced apoptosis, necrosis, and zonulin release in HK-2 cells, demonstrating the communication between them. This study found that zonulin, LPS, and LBP are potential practical markers for predicting AKI and HRS–AKI in cirrhotic patients, which may serve as potential targets for renal outcomes in cirrhotic patients.

## Introduction

Acute kidney injury (AKI) is a severe complication of cirrhosis that has a poor prognosis^1^. Recent systematic reviews reported that 29% of admitted patients with cirrhosis had AKI, whereas 10% of admitted patients with advanced cirrhosis had hepatorenal syndrome (HRS)^2,3^. AKI and HRS, especially HRS–AKI, are associated with increased mortality, the need for intensive care, and hospital readmission in patients with cirrhosis^4^. The early identification of AKI and HRS is important to the prompt management and prevention of disease progression^5,6^.

Acute tubular necrosis and injury are the major histopathological changes in cirrhotic patients with AKI and HRS^7–9^. Hypoperfusion and systemic inflammation lead to acute tubular necrosis and injury and induce the development of AKI and HRS^10,11^. The renal tubules of vulnerable areas, especially the proximal renal tubules, sustain damage^9^. The kidneys are in an ischemia–reperfusion state, which leads to systemic inflammation. This process precipitates the translocation of gut-derived endotoxins and further augments inflammation-related AKI^12^. In a rat model of renal ischemia–reperfusion, intestinal tight junction (TJ) integrity became disrupted^13^. The influx of luminal toxins induces endotoxemia and further jeopardizes renal function, the so-called gut-kidney crosstalk^14^.

TJ proteins such as occludins and zonula occludens-1 (ZO-1) transcellularly connect to maintain intact intestinal barriers and block luminal microorganisms, toxins, or allergens^15^. Intestinal epithelial cells secrete zonulin in response to inflammatory disorders^16^. Zonulin is an enterotoxin that reversibly regulates intracellular TJ^17^. When intestinal permeability is disrupted, luminal contents such as bacterial endotoxins (lipopolysaccharide, LPS) translocate from the intestine to the blood circulation through the paracellular pathway. LPS interacts with LPS-binding protein (LBP) and activates a cascade of immune responses. Activated immune cells release proinflammatory cytokines, leading to downstream systemic inflammation and organ damage^18,19^.

The pathomechanisms of intestinal barrier dysfunction are associated with various diseases, including cardiovascular diseases^20^, diabetes, obesity^21^, chronic liver diseases^22^, and kidney diseases^12^. In liver diseases, zonulin levels can predict the severity of cirrhosis and hepatic decompensation^23^. A cohort study with a mean follow-up of 16.3 ± 3.8 years reported a higher serum LPS level was associated with an increased risk of incident advanced liver disease^24^. Higher serum LBP levels were observed in deceased patients with liver cirrhosis^25^. Patients with type 2 diabetes and chronic kidney disease had higher zonulin and LPS levels than healthy individuals^26^. Another study compared hemodialysis patients with healthy controls and found that plasma zonulin levels were significantly higher in the former^27^. Despite numerous studies applying zonulin, LPS, and LBP to advanced liver disease and chronic renal disease, few studies have used them to predict AKI and HRS–AKI in patients with cirrhosis.

This prospective cohort study aimed to evaluate the roles of zonulin, LPS, and LBP in cirrhotic patients with AKI and HRS–AKI. We hypothesized that zonulin, LPS, and LBP levels increase in cirrhotic patients with AKI and HRS–AKI due to gut-kidney crosstalk. This study combined zonulin, LPS, LBP, and other clinical markers to construct predictive models for AKI and HRS–AKI. In vitro, we tested the effects of LPS and LBP on intestinal cells and the communication between intestinal cells and renal tubular cells. Through clinical and experimental evidence, we hope to provide applicable markers for health care providers to raise awareness of AKI and HRS–AKI in cirrhotic patients.

## Methods

### Study population and study design

**Figure 1:**
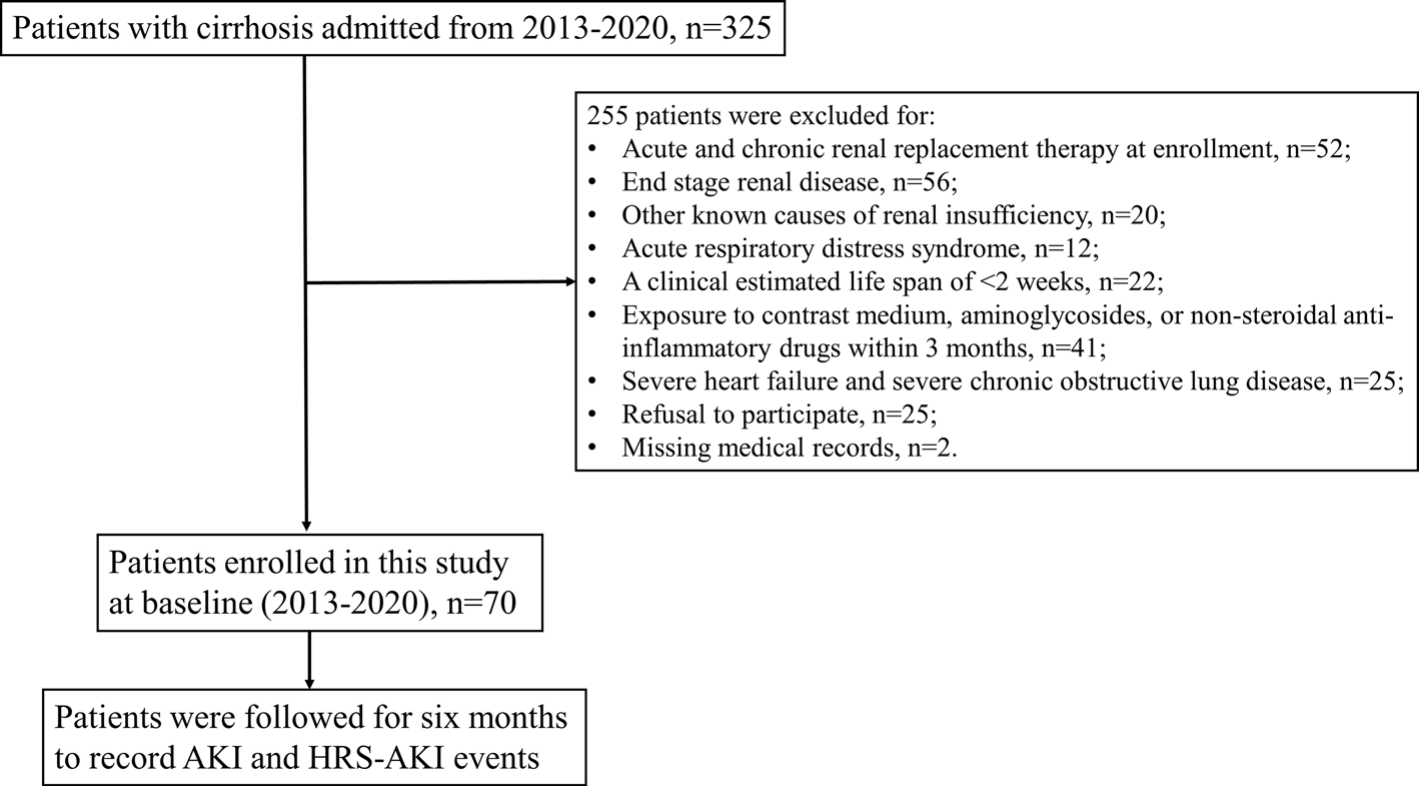
The flowchart of study design. Abbreviations: AKI, acute kidney injury; HRS, hepatorenal syndrome.

This prospective cohort study is an extension of our previous study^28^. A flowchart of the study design is shown in Fig. 1. We recruited cirrhotic patients admitted for acute hepatic decompensation in 2013–2020 at Taipei Veterans General Hospital, Taipei, Taiwan. This study was approved by the ethical committee of Taipei Veterans General Hospitals, Taipei, Taiwan (2015-09-004A, 2018-03-013AC, and 2020-06-001AC). The patients who agreed to participate signed a written informed consent form. This study was conducted in compliance with the guidelines of the Committee on Human Experimentation and the Declaration of Helsinki.

We excluded patients who met any of the following criteria: acute and chronic renal replacement therapy at enrollment, end-stage renal disease, other known causes of renal insufficiency, acute respiratory distress syndrome, a clinical estimated life span of < 2 weeks, exposure to contrast medium, aminoglycosides, or non-steroidal anti-inflammatory drugs within 3 months, severe heart failure (New York Heart Association functional class III or IV), severe chronic obstructive lung disease (Global Initiative for Chronic Obstructive Lung Disease stage 3 or 4), or missing medical records. We also confirmed that the participants were not exposed to immunosuppressants, steroids, contrast media, or other nephrotoxic drugs during follow-up.

At baseline (2013–2020), 70 patients with cirrhosis were enrolled. The patients’ basic demographic data, etiology of cirrhosis, laboratory data, and clinical events at admission (ascites, spontaneous bacterial peritonitis, encephalopathy, esophageal varices bleeding, sepsis, septic shock, acute-on-chronic liver failure) were recorded by experienced medical doctors. The physicians followed up on the medical records of the participants for six months to screen if AKI or HRS–AKI occurred. In the event of AKI or HRS–AKI, the time elapsed from the date of enrollment to the occurrence of AKI or HRS–AKI will be recorded (time to event).

### The definition of AKI, HRS–AKI, and other clinical events

We used the Kidney Disease: Improving Global Outcomes (KDIGO) guidelines to define AKI as the presence of one of the following criteria: (1) serum creatinine (sCr) increase by ≥ 0.3 mg/dL (≥ 26.5 μmol/L) within 48 h; (2) sCr increase by ≥ 50% from baseline value within the previous 1 week; and (3) decreased urine volume as ≤ 0.5 mL/kg/h for at least 6 h^29^. HRS–AKI was diagnosed according to the revised definition based on the International Club of Ascites (ICA) consensus document as follows^8^: (1) diagnosis of cirrhosis, acute liver failure, or acute-on-chronic liver failure; (2) presence of AKI according to the ICA consensus document (consistent with the KDIGO definition); (3) no response for at least 48 h of diuretic withdrawal and volume expansion with albumin by 1 g/kg body weight/day to a maximum of 100 g/day; (4) absence of shock; (5) no recent or current exposure to nephrotoxic drugs; and (6) no evidence of structural kidney disease defined as proteinuria < 500 mg/day, microhematuria (< 50 red blood cells per high-power field), normal urinary injury biomarkers (if available), and/or normal renal ultrasonography. The definitions of the other clinical events are shown in Supplementary Table S1^30,31^. All clinical events, including ascites, spontaneous bacterial peritonitis, encephalopathy, esophageal variceal bleeding, sepsis, septic shock, acute chronic liver failure, AKI, and HRS–AKI, were extracted from the patients’ medical records.

### Blood tests and other variables

The blood samples were allowed to clot and were spun immediately in a refrigerated centrifuge. The serum was obtained and frozen at -80 °C immediately**.** Blood tests including white blood cell count (WBC, /μL), platelet count (/μL), international normalized ratio (INR) based on prothrombin time, serum sodium (mEq/L), sCr (mg/dL), estimated glomerular filtration rate (eGFR, mL/min/1.73 m^2^), albumin (g/dL), total bilirubin (mg/dL), and C-reactive protein (CRP, mg/dL) were performed by automated analyzers at the central laboratory of Taipei Veterans General Hospital. LPS was determined from sterile serum samples that were diluted to 20% (vol/vol) with endotoxin-free water and then heated to 70 °C for 10 min to inactivate serum proteins. According to the manufacturer's protocol, the levels of zonulin, LPS, and LBP were examined using commercial enzyme-linked immunosorbent assay (ELISA) kits (MyBioSource, CA, USA) with sensitivity of < 0.156 ng/ml for zonulin (detection range of 0.625–40 ng/ml), with sensitivity of 0.126 ng/mL for LPS (detection range 0.32–20 ng/mL), with sensitivity of 1.875 ng/mL for LPS (detection range 3.125–200 ng/mL). For LPS level, sterile serum samples that were diluted to 20% (vol/vol) with endotoxin-free water and then heated to 70 °C for 10 min to inactivate serum proteins. We also calculated the Model for End-stage Liver Disease score incorporating sodium (MELD-Na score), Child–Pugh score, and Fibrosis-4 (FIB-4) index for each participant.

### The construction of prediction models and statistical analyses

Categorical variables between the AKI/non-AKI and HRS–AKI/non-HRS–AKI groups were compared using chi-squared tests and Fisher’s exact tests. T-tests and Mann–Whitney U tests were used to analyze continuous variables. We selected the significant risk factors for predicting AKI and HRS–AKI. The prediction model for AKI included zonulin, LPS, LBP, age, and no history of hepatitis B infection. The prediction model for HRS–AKI included zonulin, LPS, LBP, total bilirubin, and Child–Pugh score.

For continuous risk factors, receiver operating characteristic (ROC) curve analyses were performed to obtain optimal cutoff values. Continuous risk factors were categorized according to the cutoff values. The influences of categorized risk factors on AKI and HRS–AKI were analyzed by univariate Cox proportional-hazards models (Table 1). The hazard ratios were deemed as the weighted scores. We developed the prediction models for AKI and HRS–AKI by summing the weighted scores of the risk factors (Table 1). The total scores of prediction models for AKI and HRS–AKI were analyzed by ROC curve analysis. According to the optimal cutoff values in the ROC analyses, participants were grouped in both AKI and HRS–AKI prediction models. We used Kaplan–Meier curves to estimate the probability of being free from AKI and HRS–AKI and to test the differences between groups. All statistical analyses were performed using SAS version 9.4 (SAS Institute, Inc., Cary, NC, USA). Statistical significance was defined as a two-tailed *p*-value < 0.05.

**Table 1:** Univariate cox regression analyses for risk factors predicting AKI and HRS–AKI.

Risk factors	HR (95% CI)	*p*	Weight scores
AKI			
Zonulin > 7.19	26.54 (5.84–120.62)	< 0.001	26.54
LPS > 2.79	4.01 (1.23–13.01)	0.02	4.01
LBP > 83.04	5.44 (1.50–19.81)	0.01	5.44
Age > 60.99	5.44 (1.21–24.57)	0.03	5.44
CRP > 4.19	4.34 (1.46–12.92)	0.008	4.34
No hepatitis B	10.21 (2.26–46.17)	0.003	10.21
Total scores for AKI prediction model			0–55.98
HRS–AKI			
Zonulin > 7.30	40.26 (4.93–328.62)	< 0.001	40.26
LPS > 2.80	15.55 (1.91–126.49)	0.01	15.55
LBP > 83.99	11.33 (1.39–92.14)	0.02	11.33
Total bilirubin > 2	6.25 (1.26–30.97)	0.02	6.25
Child–Pugh Score > 8	4.18 (1.05–16.75)	0.04	4.18
Total scores for HRS–AKI prediction model			0–77.57

The hazard ratios were used for the scoring strategy for the prediction models of AKI and HRS–AKI. The cut points for each risk factors were obtained by receiver operating characteristic (ROC) analyses.

*AKI* acute kidney injury, *CRP* C-reactive protein, *HRS* hepatorenal syndrome, *LBP* lipopolysaccharide-binding protein, *LPS* lipopolysaccharide.

### In vitro analyses-cell line and culture conditions

The human colon carcinoma cell line, Caco-2, was obtained from the American Type Culture Collection (ATCC, Rockville, USA) and maintained in Dulbecco’s modified Eagle’s medium (DMEM; Lonza Benelux BV, Breda, The Netherlands) containing 4.5 g/L glucose, L-glutamine, 10% (v/v) fetal calf serum (Invitrogen, Breda, The Netherlands), 1% (v/v) non–essential amino acids (Invitrogen), 1% (v/v) antibiotic/antimycotic mixture (10,000 units of penicillin, 10,000 µg of streptomycin, and 25 µg/mL of amphotericin B; Invitrogen). Human kidney 2 (HK-2) cells, an immortalized human kidney proximal tubular epithelial cell line, was purchased from ATCC. HK-2 cells were grown in DMEM containing 10% fetal bovine serum and 1% penicillin & streptomycin. Cells were maintained at 37 °C in 5% CO_2_ atmosphere. The experiment was repeated three times. Lyophilized LPS powder was purchased from Sigma-Aldrich (St. Louis, MO, *USA*). Human recombinant LBP (hrLBP) was purchased from R&D Systems (Minneapolis, MN). AT1001 (larazotide, Biopolymer Laboratories, University of Maryland, Baltimore, MD) is a zonulin synthetic peptide antagonist that able to block its effects was dissolved in DMSO without up to 3–5 mM^32^.

Culture Caco-2 cells were seeded into 96-well plates at a density of 5000 cells per well. Firstly, we evaluated the effects of LPS, hrLBP, and LPS+hrLBP on apoptosis and necrosis (by terminal deoxynucleotidyl transferase dUTP nick end labeling [TUNEL] stain, Caspase-3 stain, and Annexin V/PI stain) in Caco-2 cells. The zonulin release and the expressions of occludin and ZO-1 in lysates of Caco-2 cells were also measured.

It had been reported that 1 μM of AT1001 can blocked the ischemia-induced TJ dysfunction but higher concentration (10 μM) of AT1001 did not elicit better protective effects^32,33^. So, in preliminary experiments (n = 3 in each group), the dose-dependent effects of 5 μM, 10 μM, 20 μM of AT1001 for the inhibition of LPS-induced zonulin release in supernatant of Coca2 cells were tested. The results showed that the degree of the inhibition of LPS-induced zonulin release were higher in 10 μM-AT1001 group (decrease from 1.2 to 0.8 ng/mL, 33% of decrease) than in 5 μM-AT1001 group (decrease from 1.2 to 1.04 ng/mL, 16% of decrease). However, the 20 μM-AT1001 group (decrease from 1.2 to 0.78 ng/mL, 35% of decrease) did not induce further decrease in LPS-induced zonulin release. Accordingly, 10 μM of AT100 was used in serial of experiments.

In order to evaluate the roles of zonulin in the LPS+hrLBP-induced apoptosis and necrosis in Caco-2 cells. The abovementioned experiments were repeated after 24 h pre-treatment with AT1001 (larazotide, zonulin antagonist) on LPS+hrLBP group of Caco-2 cells.

To detect the communications between Caco-2 cells and HK-2 cells, we collected the supernatant of various pre-treated Caco-2 cells as conditioned medium (CM). HK-2 cells were incubated with CM (10^−5^ M) for 24 h. The responses of HK-2 cells were recorded, including apoptosis, necrosis, and release of lactate dehydrogenase (LDH, cell injury markers). We also evaluated the epithelial-mesenchymal transition (EMT) and migration of HK-2 cells because both are important pathogenesis for renal interstitial fibrosis. HK-2 cells were incubated with transforming growth factor (TGF)-β1 (2 ng/mL) which is a key factor for the EMT. Then the EMT and migration of HK-2 cells were recorded. The EMT was evaluated by the downregulated mRNA expression of epithelial marker (E-cadherin) and the upregulated expression of mesenchymal markers (α-SMA and vimentin). Subsequently, HK-2 cells were treated with TGF-β1 plus CM from pre-treated Caco-2 cells to observe if the TGF-β1-induced EMT and migration of HK-2 cells were modified. For q-RT-PCR, Total RNA was purified from cell lysates using TRIzol reagent (Invitrogen) and an RNeasy Mini kit (Qiagen). The quantity and quality of the RNA were determined spectroscopically using a nanodrop (Thermo Scientific). Total RNA was used to synthesize cDNA using the Transcriptor cDNA First-Strand Synthesis Kit (Roche) according to the manufacturer’s protocol and was resuspended in 50 λ of H2O. cDNA samples (2λ) were used for real-time PCR in a total volume of 25 λ using SYBR Green Reagent (Invitrogen) and specific primers (Table 1) on a qPCR machine (Applied Biosystems 7300 Sequence Detection System). All real-time PCRs were performed in duplicates. Data were generated and analyzed using SDS 2.3 and RQ manager 1.2 software. The samples’ threshold cycle (Ct) values were computed, a script levels were determined using the 2 − ΔCt method and the expression values were normalized to the level of 18S mRNA. All primers listed in Supplementary Table  S2 were purchased from Applied Biosystems.

For western blot analysis, cells were homogenized in a lysis buffer supplemented with protease inhibitors (1 mM phenylmethylsulfonyl fluoride; 100 mM; 2 g/ml aprotinin, 2 g/ml leupeptin), the supernatant was collected and protein concentrations in cell lysates were determined by the Pierce™ BCA Protein Assay Kit with Bradford reagent (Thermo Fisher Scientific Inc., IL, USA). Cell lysate were added at 0 °C to 10 ml of variables in 23 assay mix [1 mM ATP, 20 mM DTT, 0.4 mM IBMX, and 4 mM of either MgCl2 (Mg21 assay mix) or MnCl2 (Mn21 assay mix) in LB]. Reactions were initiated by transferring the samples to a 23 °C water bath and were terminated by addition of 10 ml 0.4 M EDTA and by boiling the samples for 1 min. The ZO-1/occludin /claudin-1 [purchased from Invitrogen Corporation (Carlsbad, CA, USA) and Sigma (St. Louis, MO, USA.), and Abcam [(Cambridge, MA, USA)] and secondary antibody were applied to each polyvinylidene fluoride membranes. Image J software was used to assess the density of the protein of the interest band, which was then normalized to the GADPH protein band.

### Apoptosis assay

An in situ apoptosis detection (Chemicon, CA, USA) and an Annexin V/PI assay kit were used to measure the percentage of apoptotic and necrotic (TUNEL+ or Annexin V+/PI+, respectively) cells in the cell population. Using a colorimetric CaspACE assay system (Promega), total proteins were extracted and quantified, and equal amounts (50 µg) of proteins were used in the assay. In the assay system, the colorimetric substrate (N-Acetyl-Asp-Glu-Val-Asp p-nitroanilide, Ac-DEVD-pNA) is labeled with the chromophore *p*-nitroaniline (pNA). pNA is released from the substrate upon cleavage by caspase-3. Free pNA produces a yellow color that is measured using a plate reader at 450 nm after 18 h of incubation at 37 °C. The amount of yellow color produced upon cleavage is proportional to the amount of caspase-3 activity presents in the samples. For each assay, in addition to the samples, the following standards were also tested: (1) a blank consisting of assay buffer and substrate only (to verify that the coloration was not due to substrate degradation over time), (2) a negative control containing caspase 3 inhibitor (to determine the signal baseline), and (3) a positive control containing commercial caspase 3 provided with the kit (to verify of efficacy of the test). Caspase-3 enzymatic activity in cell lysates of Caco-2 cells was determined using a colorimetric assay (CaspACE assay system, Promega). The data were normalized to the protein content in each sample.

### Evaluation of cell necrosis by lactate dehydrogenase

Leakage of LDH into the culture medium of the HK-2 cell culture system reflects compromised cell membrane integrity, indicating necrosis severity. To evaluate HK-2 cell necrosis, the culture medium was collected and centrifuged at 3000 rpm for 5 min to obtain cell-free supernatant, while LDH in the medium was measured using a Cayman chemical kit. Samples were diluted in assay buffer and combined with nicotinamide adenine dinucleotide (NAD^+^), lactic acid, tetrazolium salt, and diaphorase. Briefly, extracellular LDH catalyzes the conversion of lactate to pyruvate via the reduction of NAD^+^ to its reduced form (NADH). Diaphorase then uses NADH to reduce the tetrazolium salt to a red formazan product. The absorbance was read at 490 nm, and results were expressed as the percentage of LDH release in samples relative to that in the buffer group after deduction of the absorbance value at 680 nm (background signal).

### EMT-related cell migration assay

To assess another aspect of the EMT in HK-2 cells, the TGF-β1-induced cell migration assay was performed. HK-2 cell migration was assessed using a chemotaxis chamber with 0.3 cm^2^ inserts of 8-mm pore membranes placed in 12-well culture dishes to form upper and lower compartments (Transwell, Corning Costar, Cambridge, MA, USA). The lower compartment contained medium (buffer) alone (DMEM/0.2% bovine serum albumin) or medium with TGF-β1 (2 ng/mL) plus test agents and pre-coated with type I collagen (50 mg/L). The upper compartment was seeded with HK-2 cells (3–3.5 × 10^5^ cells/mL in 150 μL of serum-free medium) that were serum-starved for 24 h. The entire chamber was incubated at 37 °C for 24 h to allow cell migration. At the end of the experiments, HK-2 cells remaining on the upper surface of the filters were removed using cotton tips. The membranes in the lower compartment containing migrated cells were fixed in 100% methanol, stained with 0.1% crystal violet, mounted in glycerol on glass slides, and examined under a microscope. The migrated HK-2 cells were counted in 10 random high-power fields (630 × magnification) and expressed as migrating cells/field of view. Additionally, in order to evaluate the roles of zonulin in Caco-2-HK-2 cells communication, CM of LPS+hrLBP+AT1001 group of Caco-2 cells on the apoptosis, necrosis, EMT and migration of HK-2 cells were measured and compared with CM of LPS+hrLBP group of Caco-2 cells-related effects.

## Results

### The demographic and clinical characteristics

Significantly, the Pearson correlation analyses (*r* = 0.304, *p* = 0.01) show serum Cr and LBP are moderate positive correlated (*p* < 0.05) in all cirrhotic patients. The correlations between Child–Pugh Score and LPS (*r* = 0.39, *p* = 0.001) and Zonulin (r = 0.36, *p* = 0.003) show significantly moderate positive correlation. There is also a trend for moderate positive correlation between Child–Pugh Score and LBP (r = 0.22, *p* = 0.07) and Zonulin (r = 0.36, *p* = 0.003). The correlations between Albumin-Bilirubin (ALBI) Grade and LPS (r = 0.37, *p* = 0.002) and Zonulin (r = 0.24, *p* = 0.04) show significantly moderate positive correlation. During the follow-up period, 13 (18.6%) and eight (11.4%) cirrhotic patients developed AKI and HRS–AKI, respectively. Compared with the non-AKI group, patients with AKI were older (mean age 71.1 ± 12.0 vs. 59.2 ± 11.2 years) and less likely to have hepatitis B (15.4% vs. 70.2%). AKI patients also had higher CRP levels (5.2 ± 4.2 vs. 3.4 ± 4.9 mg/dL), zonulin levels (7.1 ± 1.7 vs. 4.7 ± 1.4 ng/mL), LPS levels (3.2 ± 1.3 vs. 2.2 ± 0.9 ng/mL), and LBP levels (92.2 ± 20.2 vs. 72.7 ± 21.3 ng/mL). Cirrhotic patients with HRS–AKI had higher levels of total bilirubin (3.3 ± 2.1 vs. 3.1 ± 6.4 mg/dL), zonulin (7.9 ± 0.4 vs. 4.8 ± 1.6 ng/mL), LPS (3.7 ± 0.9 vs. 2.3 ± 1.0 ng/mL), and LBP (94.6 ± 14.7 vs. 74.0 ± 22.0 ng/mL) as well as a higher mean Child–Pugh score (8.4 ± 1.4 vs. 7.2 ± 1.9) than those without HRS–AKI. There were no significant intergroup differences in sex, WBC count, platelet count, INR, serum sodium, sCr, eGFR, albumin, MELD-Na score, FIB-4 index, or clinical events at admission (Tables 2 and 3).

**Table 2:** The demographic and clinical characteristics of cirrhotic patients with and without AKI during the six-month follow-up period.

	AKI	Non-AKI	Total	*p*
	n = 13 (18.6%)	n = 57 (81.4%)	n = 70	
Age (mean ± SD)	71.1 (12.0)	59.2 (11.2)	61.4 (12.2)	0.001
Male, n (%)	9 (69.2)	48 (84.2)	57 (81.4)	0.24
Etiology of cirrhosis, n (%)				
Hepatitis B	2 (15.4)	40 (70.2)	42 (62)	< 0.001
Hepatitis C	5 (38.5)	16 (28.1)	21 (30)	0.51
Alcohol	5 (38.5)	20 (35.1)	25 (35.7)	1
Others	3 (23.1)	2 (3.5)	5 (7.1)	0.04
Laboratory data (mean ± SD)				
White blood cell count (/μL)	5823.1 (2396.2)	4736.3 (1734.2)	4938.1 (1902.8)	0.22
Platelet count (/μL)	136,384.6 (110,508.3)	101,403.5 (51,916.4)	107,900 (67,075.2)	0.56
INR	1.2 (0.2)	1.3 (0.3)	1.3 (0.2)	0.84
Sodium (mEq/L)	137.4 (4.9)	136.8 (3.8)	136.9 (4.0)	0.35
sCr (mg/dL)	0.9 (0.3)	1 (0.6)	1 (0.5)	0.85
eGFR (mL/min/1.73 m^2^)	80.2 (41.0)	90.4 (31.1)	88.9 (32.6)	0.37
Albumin (g/dL)	3.2 (0.6)	3.3 (0.6)	3.3 (0.6)	0.86
Total bilirubin (mg/dL)	2.4 (2.0)	3.2 (6.7)	3.1 (6.1)	0.35
C-reactive protein (mg/dL)	5.2 (4.2)	3.4 (4.9)	3.7 (4.8)	0.03
MELD-Na score	10.9 (7.1)	9.9 (8.8)	10.1 (8.5)	0.43
Child Pugh score	7.8 (1.7)	7.2 (1.9)	7.3 (1.9)	0.16
FIB-4 index	10.7 (7.2)	10.4 (15.0)	10.4 (14.1)	0.33
Zonulin (ng/mL)	7.1 (1.7)	4.7 (1.4)	5.1 (1.8)	< 0.001
LPS (ng/mL)	3.2 (1.3)	2.2 (0.9)	2.4 (1.1)	0.02
LBP (ng/mL)	92.2 (20.2)	72.7 (21.3)	76.3 (22.3)	0.004
Clinical events at admission, n (%)				
Ascites	8 (61.5)	22 (38.6)	30 (42.9)	0.21
Spontaneous bacterial peritonitis	1 (7.7)	4 (7.0)	5 (7.1)	1
Encephalopathy	1 (7.7)	4 (7.0)	5 (7.1)	1
Esophageal varices bleeding	0	6 (10.5)	6 (8.6)	0.58
Sepsis	4 (30.8)	5 (8.8)	9 (12.9)	0.05
Septic shock	0	3 (5.3)	3 (4.3)	1
Acute on chronic liver failure	3 (23.1)	13 (22.8)	16 (22.9)	1

Chi-squared tests and Fisher’s exact tests were used for categorical variables. T-tests and Mann–Whitney U tests were used for continuous variables.

*AKI* acute kidney injury, *eGFR* estimated glomerular filtration rate, *FIB-4 index* Fibrosis-4 index, *HRS* hepatorenal syndrome, *INR* international normalized ratio, *LBP* lipopolysaccharide-binding protein, *LPS* lipopolysaccharides, *MELD-Na score* model for end-stage liver disease score incorporating sodium, *sCr* serum creatinine, *SD* standard deviation.

**Table 3:** The demographic and clinical characteristics of cirrhosis patients with and without HRS–AKI during the six-month follow-up period.

	HRS–AKI	Non-HRS–AKI	*p*
	n = 8 (11.4%)	n = 62 (88.6%)	
Age (mean ± SD)	65.9 (7.6)	60.8 (12.6)	0.27
Male, n (%)	6 (75.0)	51 (82.3)	0.64
Etiology of cirrhosis, n (%)			
Hepatitis B	2 (25)	40 (64.5)	0.05
Hepatitis C	2 (25)	19 (30.7)	1
Alcohol	5 (62.5)	20 (32.3)	0.12
Others	1 (12.5)	4 (6.5)	0.47
Laboratory data (mean ± SD)			
White blood cell count (/μL)	5025 (1887.4)	4926.9 (1919.8)	0.95
Platelet count (/μL)	92,875 (45,460.9)	109,838.7 (69,415.6)	0.62
INR	1.3 (0.1)	1.3 (0.3)	0.26
Sodium (mEq/L)	135.8 (5.5)	137.1 (3.8)	0.64
sCr (mg/dL)	0.97 (0.40)	1.0 (0.6)	0.93
eGFR (mL/min/1.73 m^2^)	90.6 (45.8)	88.7 (31.3)	0.89
Albumin (g/dL)	3.2 (0.7)	3.3 (0.5)	0.52
Total bilirubin (mg/dL)	3.3 (2.1)	3.1 (6.4)	0.01
C-reactive protein (mg/dL)	5.1 (4.1)	3.5 (4.8)	0.06
MELD-Na score	11.9 (5.8)	9.9 (8.8)	0.29
Child Pugh score	8.4 (1.4)	7.2 (1.9)	0.04
FIB-4 index	10.8 (8.3)	10.4 (14.7)	0.55
Zonulin (ng/mL)	7.9 (0.4)	4.8 (1.6)	< 0.001
LPS (ng/mL)	3.7 (0.9)	2.3 (1.0)	0.001
LBP (ng/mL)	94.6 (14.7)	74.0 (22.0)	0.01
Clinical events at admission, n (%)			
Ascites	6 (75)	24 (38.7)	0.07
Spontaneous bacterial peritonitis	1 (12.5)	4 (6.5)	0.47
Encephalopathy	1 (12.5)	4 (6.5)	0.47
Esophageal varices bleeding	0	6 (9.7)	1
Sepsis	3 (37.5)	6 (9.7)	0.06
Septic shock	0	3 (4.8)	1
Acute on chronic liver failure	2 (25)	14 (22.6)	1

Chi-squared tests and Fisher’s exact tests were used for categorical variables. T-tests and Mann–Whitney U tests were used for continuous variables.

*AKI* acute kidney injury, *eGFR* estimated glomerular filtration rate, *FIB-4 index* Fibrosis-4 index, *HRS* hepatorenal syndrome, *INR* international normalized ratio, *LBP* lipopolysaccharide-binding protein, *LPS* lipoptolysaccharides, *MELD-Na score* model for end-stage liver disease score incorporating sodium, *sCr* serum creatinine, *SD* standard deviation.

### The prediction models predicted AKI and HRS–AKI

**Figure 2:**
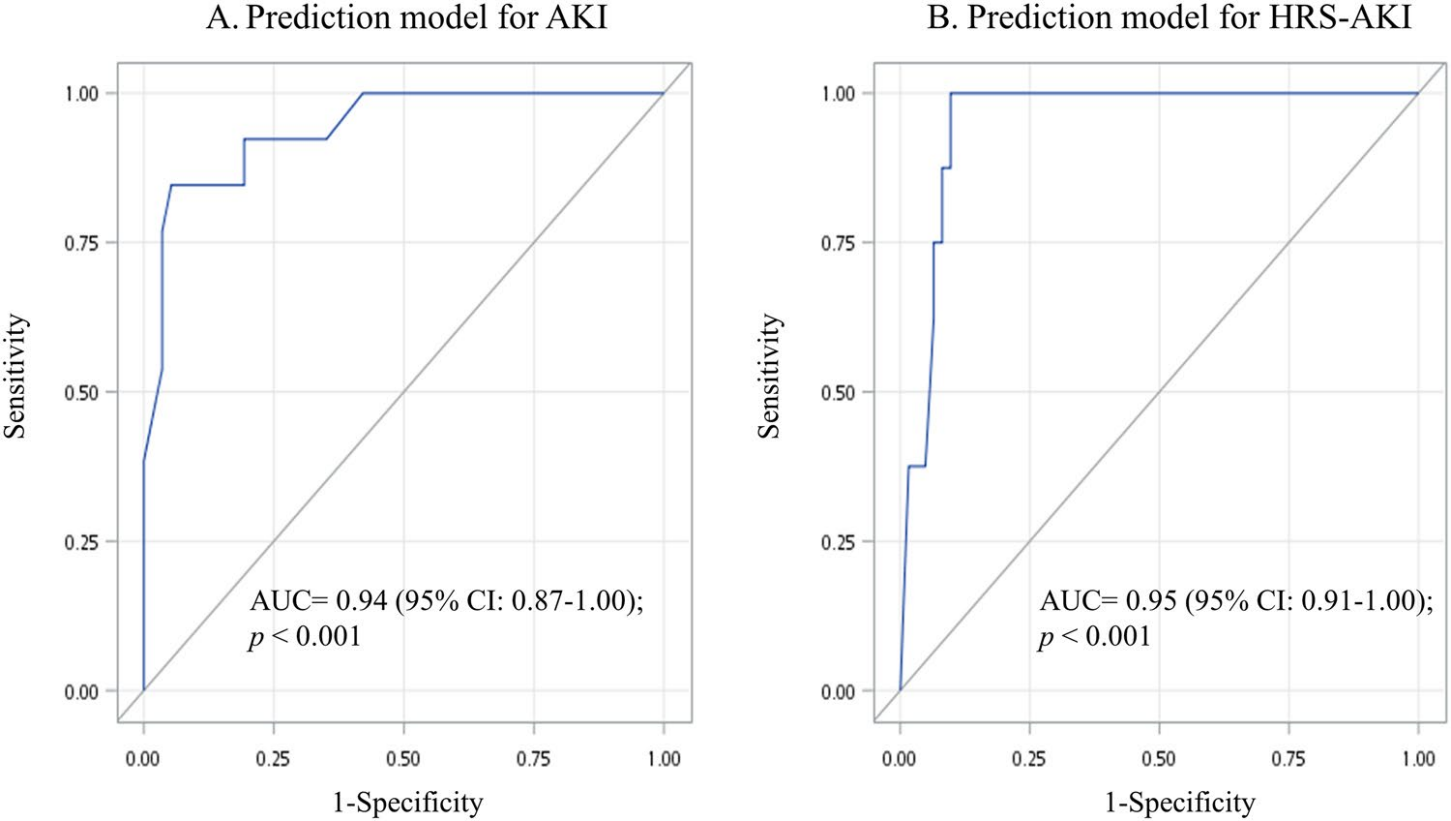
The receiver operating characteristic (ROC) analyses for the prediction models of AKI and HRS–AKI. (**A**) Prediction model for AKI included zonulin, LPS, LBP, C-reactive protein, age, and no hepatitis B; (**B**) Prediction model for HRS–AKI included zonulin, LPS, LBP, total bilirubin, and Child–Pugh Score. Abbreviations: AKI, acute kidney injury; HRS, hepatorenal syndrome; LBP, lipopolysaccharide-binding protein; LPS, lipopolysaccharides.

The risk score for AKI was 0 to 55.98. The area under curve (AUC) was 0.94 (95% confidence interval [CI]: 0.87–1.00; *p* < 0.001, Table 1 and Fig. 2). The optimal cutoff value was ≥ 40.33 with a sensitivity of 84.6% and specificity of 94.7%. The risk score for HRS–AKI was 0–77.57. The AUC was 0.95 (95% CI 0.91–1.00; *p* < 0.001). The optimal cutoff value of prediction scores for HRS–AKI was ≥ 37.30, which yielded a sensitivity of 100% and specificity of 90.3%.

**Figure 3:**
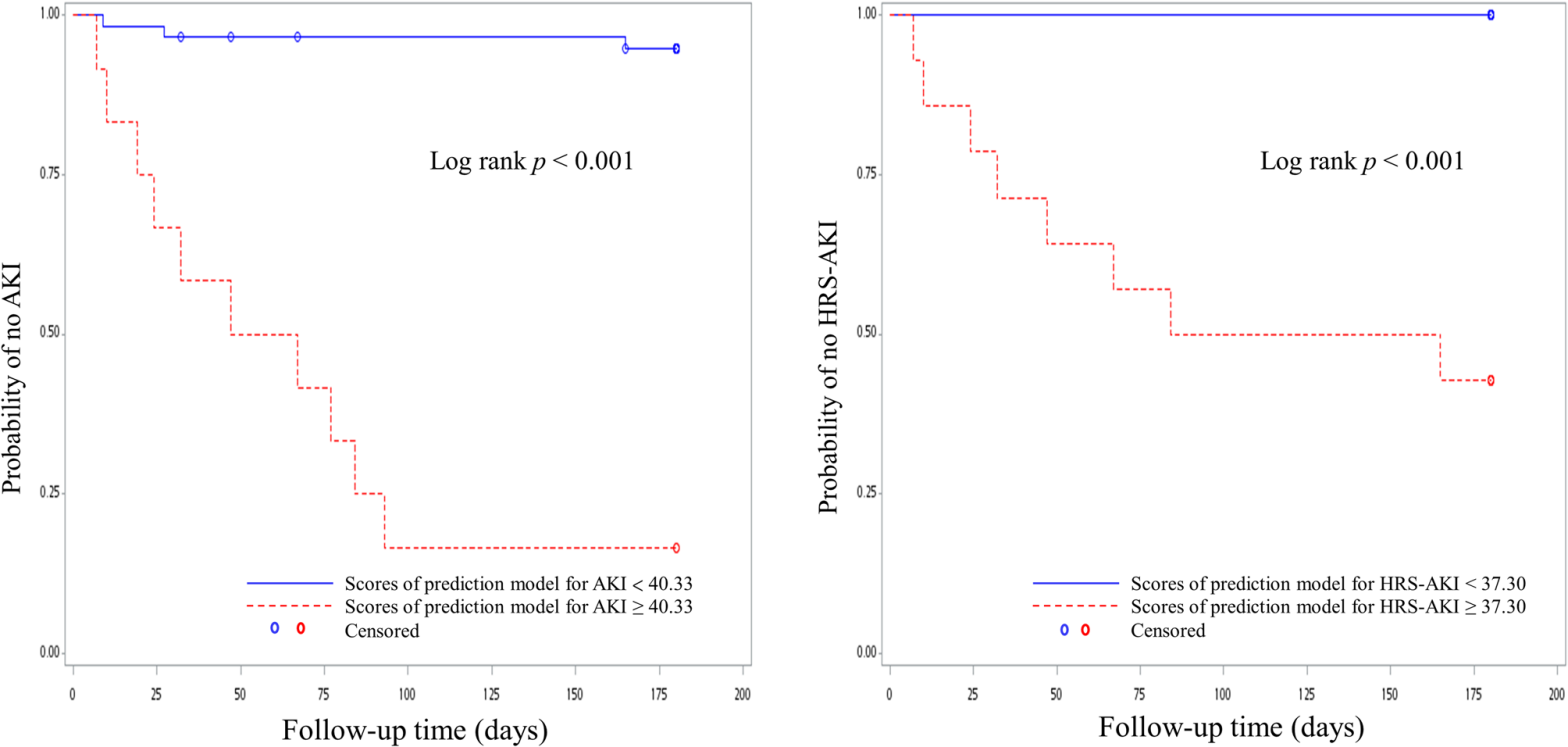
Kaplan–Meier curves displaying the estimated probability of no AKI and HRS–AKI. The cutoff values for categorization of both prediction models were obtained from receiver operating characteristic (ROC) analyses.

In Kaplan–Meier analyses, the estimated probability of being free from AKI and HRS–AKI were significantly different between high-score group and low-score group in both AKI and HRS–AKI prediction models (log-rank test *p* < 0.001, Fig. 3).

### In vitro experiments-LPS and hrLBP induced apoptosis and necrosis in Caco-2 cells

**Figure 4:**
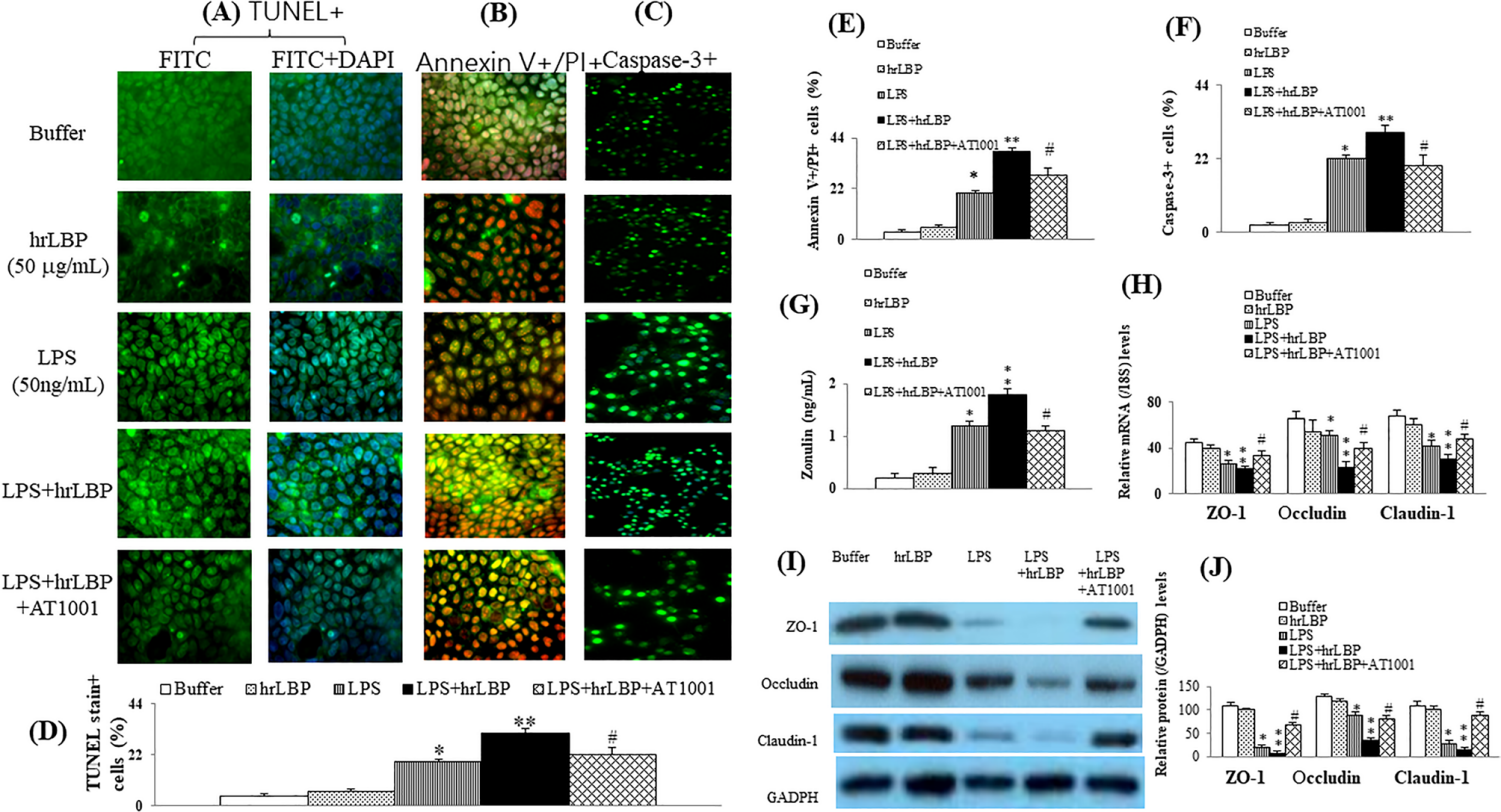
Tunnel stain+cells (%) in Caco-2 cell monolayers (**A**: immunofluorescence image, 630 × and **D**: bar graph); Annexin V+/PI+cells in Caco-2 cell monolayers (**B**: immunofluorescence image and **E**: bar graph); Caspase-3+ cells in Caco-2 cell monolayers (**C**: immunofluorescence image and **F**: bar graph); Levels of zonulin (G), relative mRNA levels of TJ proteins (ZO-1, occludin and claudin-1) (**H**), and representative images and bar graph of relative protein levels of TJ proteins (ZO-1, occludin and claudin-1) (**I**,**J**) in the supernatant of Caco-2 cells. *Note:* *,**p < 0.05 and 0.01 versus buffer group; #*p* < 0.05 versus LPS+hrLBP group. Abbreviations: DAPI, 4’,6-diamidino-2-phenylindole; FITC, Fluorescein isothiocyanate; hrLBP, human recombinant lipopolysaccharide-binding protein; LPS, lipopolysaccharides; TUNEL, terminal deoxynucleotidyl transferase dUTP nick end labeling; ZO-1, zonula occludens-1.

Figure 4 shows the apoptosis and necrosis of Caco-2 cells, release of zonulin, and expressions of TJ proteins (ZO-1/occluding/claudin-1) under different incubation conditions. Compared to the buffer group, hrLBP did not induce significant apoptosis and necrosis in Caco-2 cells. However, mild and severe apoptosis associated with Caco-2 cellular necrosis were observed in the LPS and LPS+hrLBP groups, respectively. The effect of LPS+hrLBP was attenuated by co-incubation with AT1001 (zonulin antagonist) (Fig. 4A–F). Figure 4G–J showed zonulin release, and TJ proteins (ZO-1/occluding/claudin-1) downregulation in the cell lysates from Caco-2 cells were strongest in the LPS+hrLBP group and concomitantly inhibited by AT1001(zonulin antagonist). These results suggest that LPS+hrLBP-related cellular apoptosis and necrosis were accompanied by the release of zonulin and downregulation of TJ proteins expression in Caco-2 cells.

### The communications between Caco-2 cells4 and HK-2 cells

**Figure 5:**
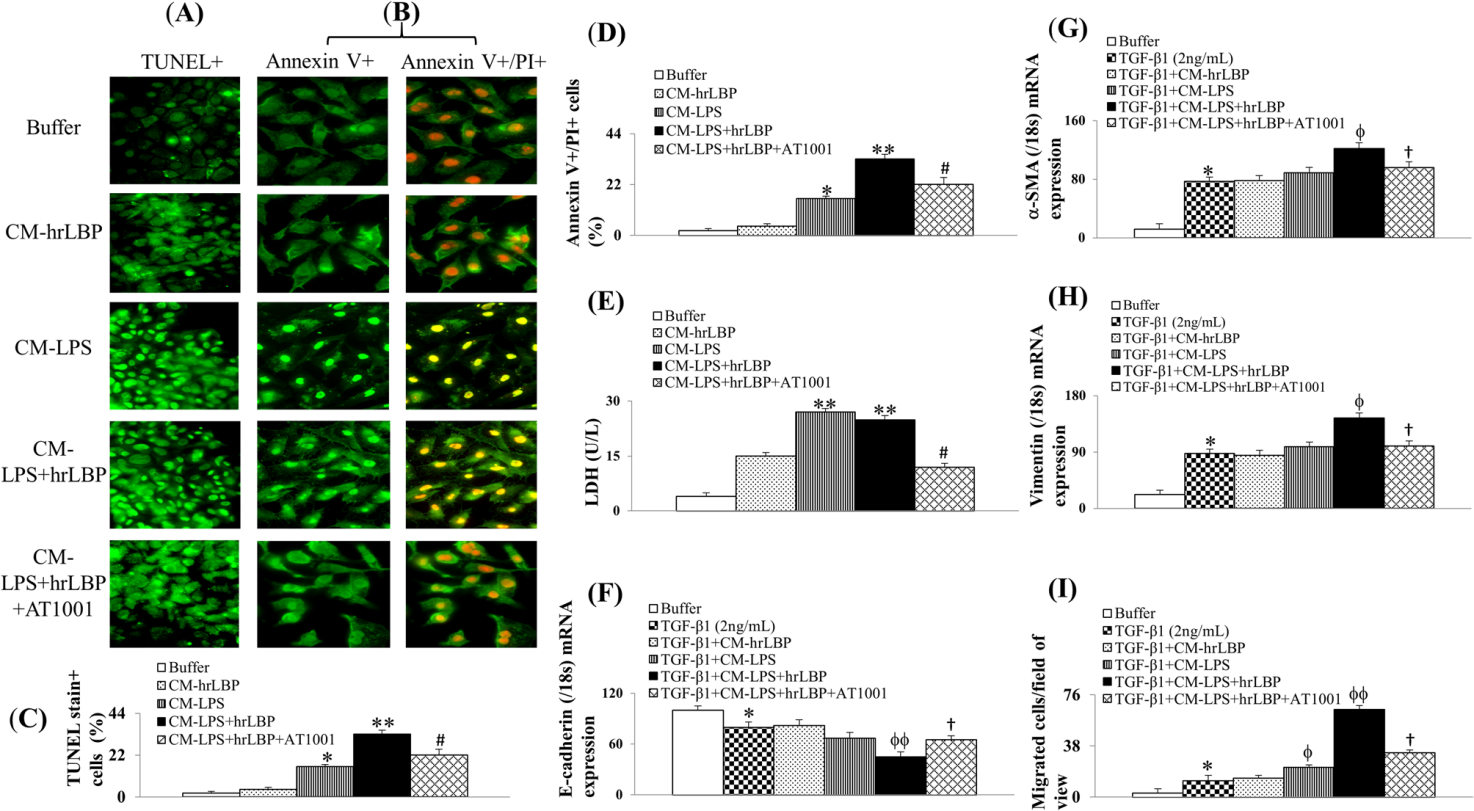
HK-2 cell monolayers were incubated with CM from various pre-treated Caco-2 cells, which were labeled by TUNEL stain (**A**,**C**) and Annexin V/PI stain (**B**,**D**); Release of LDH in the supernatant of HK-2 cell monolayers after incubation with CM from various pre-treated Caco-2 cells (**E**); TGF-*β*1 induced downregulation of epithelial marker (E-cadherin) and upregulation of interstitial markers (α-SMA and vimentin) in HK-2 cells, modified by CM from pre-treated Caco-2 cells (**F**–**H**); The count of migrated cells per field of view in response to various CM from pre-treated Caco-2 cells (**I**). *Note:* *, **p < 0.05 and 0.01 versus buffer group; #*p* < 0.05 versus CM-LPS+hrLBP group; ϕ, ϕϕ*p* < 0.05 or 0.01 versus TGF-β1 group; †*p* < 0.05 versus TGF-β1+CM-LPS+hrLBP group. Abbreviations: CM, conditioned medium; HK-2, human kidney 2; hrLBP, human recombinant lipopolysaccharide-binding protein; LDH, lactate dehydrogenase; LPS, lipopolysaccharides; TGF, tumor growth factor; TUNEL, terminal deoxynucleotidyl transferase dUTP nick end labeling.

The CM from CaCo2 cells pre-treated with hrLBP did not induce significant apoptosis and necrosis in HK-2 cells (Fig. 5A–E). However, mild apoptosis and necrosis of HK-2 cells were observed in the CM-LPS group, effects that were more severe in the CM-LPS+hrLBP group. AT1001 (zonulin antagonist) attenuated the interactive effects of CM from LPS+hrLBP-pretreated Caco-2 cells on the apoptosis and necrosis of HK-2 cells.

TGF-β1 induced EMT in HK-2 cells (Fig. 5F–H). Their co-incubation with CM from pre-treated Caco-2 cells exhibited the strongest effects of EMT in the CM-LPS+hrLBP group, including downregulation of the epithelial marker E-cadherin (Fig. 5F), upregulation of the interstitial markers α-SMA and vimentin (Fig. 5G,H), and enhanced migration (Fig. 5I). Consistent with previous experiments, AT1001 (zonulin antagonist) attenuated these responses.

## Discussion

This study found that zonulin, LPS, and LBP are reliable markers for AKI and HRS–AKI in patients with cirrhosis. We developed combined predictive models to predict AKI and HRS–AKI for application in clinical practice. We also proved that zonulin, LPS, and LBP mediate toxic reactions in intestinal cells and are involved in the interactions between intestinal cells and renal tubular cells, supporting the clinical findings.

The occurrences of AKI and HRS–AKI in patients with cirrhosis usually implies marked inflammation and a poor prognosis. Both serum CRP and hypersensitive CRP (hs-CRP) will increase in response to inflammation. The sensitivity of the hs-CRP test detects small increases of CRP in the bloodstream and allows to detect slightly elevated CRP levels that would otherwise go unnoticed with a regular CRP test^34^. In this study, compared with the non-AKI group, cirrhotic patients with AKI had significantly higher CRP levels. Accordingly, in addition to CRP, hs-CRP should be measured in future study to emphasize the link between inflammation and AKI.

Previous studies attempted to explore markers for the early identification of AKI in cirrhotic patients. Gameiro et al*.* analyzed 186 patients with cirrhosis in Portugal and reported that AKI patients had higher sCr levels, neutrophil-to-lymphocyte ratio, and modified MELD-Na score than those without AKI. Gameiro et al*.*^35^ developed a prediction score for AKI that incorporated the above variables, which yielded an AUC of 0.86 (95% CI 0.803–0.908), sensitivity of 88.5%, and specificity of 72.4%. However, sCr may be falsely low in cirrhotic patients because of muscle wasting and decreased hepatic Cr synthesis^36^, which may lower the model’s predictive power. Jo et al*.*^37^ investigated 111 hospitalized patients with cirrhosis in Korea who had stable renal function before admission. They found that plasma urine neutrophil gelatinase-associated lipocalin was sensitive for predicting early AKI upon admission. The AUC was 0.707 (95% CI 0.604–0.797), with a sensitivity of 47.73% and specificity of 92%. They concluded that renal tubular injury markers were better able to detect AKI than renal functional markers such as sCr.

This study used markers involved in leaky gut mechanisms, including zonulin, LPS, and LBP, to predict AKI and HRS–AKI in patients with advanced cirrhosis. These markers may be more sensitive than renal injury markers because gut-kidney crosstalk is crucial to the pathogenesis of renal injury. Markers representing the severity of inflammation (CRP) and cirrhosis (total bilirubin and Child–Pugh score) were also important for AKI and HRS–AKI and combined in the prediction models. Compared with previous studies, we did not use the sCr and MELD-Na scores in the models because these markers were similar between the AKI/non-AKI and HRS–AKI/non-HRS–AKI groups in this study, lacking good predictive power.

Apoptosis, necrosis and zonulin release are important mechanisms for disruption of intestine mucosa barrier^38,39^. In CD patients, the gliadin-induced zonulin release and subsequent increased intestinal permeability can be blocked by the zonulin antagonist AT-1001^39^. Our in vitro analyses showed that apoptosis, necrosis, and zonulin secretion by intestinal cells were triggered by LPS and even stronger under LPS+hrLBP exposure. The CM from the pre-treated intestinal cells had toxic effects on renal tubular cells, indicating an interaction between them that involved LPS, LBP, and zonulin. LPS results in barrier dysfunction by stimulating inflammatory cytokines^40^. Asmar et al*.*^41^ showed that zonulin secretion is stimulated by bacteria in mammalian small intestinal cells. Our results were also in accordance with those of a previous report that LPS induces zonulin secretion in Caco-2 cells^42^. LBP can deliver LPS to immune cells and activate the immune response^43^. Thus, our study demonstrated a synergistic effect of LPS and LBP on zonulin secretion and cellular apoptosis. Notably, the physiological role of zonulin is a double-edged sword. The secretion of zonulin in response to microorganisms induces TJ opening and water secretion into the gut lumen to flush out microorganisms. Under pathological conditions, zonulin is inappropriately activated, leading to an unregulated open paracellular pathway for microorganisms and endotoxemia^44^.

EMT is the response to cell injury that transforms epithelial cells into mesenchymal cells and accumulates fibrosis. Renal tubular atrophy and chronic kidney disease develop when renal tubular cells undergo EMT. EMT occurs upon stimulation by TGF-β1 and other inflammatory activators^45^.

Previous studies reported that LPS can induce EMT in intrahepatic biliary and pulmonary alveolar epithelial cells^46,47^. Our in vitro study showed that CM from LPS+hrLBP-treated Caco-2 cells induced EMT in HK-2 cells. The results indicated LPS, LBP, zonulin, and other signals released from pre-treated intestinal cells contributed to EMT in renal tubular cells. Our results provide evidence of the communication between intestinal cells and renal tubular cells, which may support the pathomechanism of gut-kidney crosstalk.

Pathophysiologically, therapies that ameliorate intestinal barrier were potential targets for reducing inflammatory stress and improving outcomes of advanced cirrhosis. In animal models, obeticholic acid normalizes TJ protein expression and decreases bacterial translocation in cirrhotic rats^48^. In another cirrhotic rat model, pioglitazone improved endotoxemia-induced renal dysfunction^49^. A case–control study in humans reported that rifaximin decreased the incidence of AKI and HRS in 88 patients with cirrhosis by inhibiting intestinal bacterial overgrowth and translocation^50^. Zonulin inhibitors have been studied for their therapeutic use in various inflammatory diseases such as celiac disease, type 1 diabetes, autoimmune diseases, and inflammatory bowel diseases^51^. This study suggests that zonulin and LBP are potential targets of renal outcomes in decompensated cirrhosis. Future research is warranted to explore the pharmacologic therapy aim at these markers and the effects on renal function in patients with cirrhosis.

This study had some limitations. First, we used hrLBP instead of extracting LBP from patients’ serum in the in vitro analyses, which may have led to missing of the complex immune substances in cirrhotic patients. Because molecular activity is easily lost during the preservation of human serum, we used hrLBP to determine its effects on intestinal cells. Second, the sample size was small and its statistical power may have been insufficient. A small sample size tended to lead to a null effect, while we still observed the significance of zonulin, LPS, and LBP. Third, the study population was recruited from a medical center in northern Taiwan. For conclude the observation from clinical data about the roles of serum zonulin, LPS, and LBP for predicting AKI and HRS–AKI in cirrhotic patients, future systematically cell line work is necessary. The prediction models created in this study lacks validation testing for other populations. We demonstrated the essential roles of zonulin, LPS, and LBP in renal outcomes of patients with cirrhosis. Based on this evidence, future studies can use the prediction models for further testing. Finally, in vitro experiments may not fully represent the complex interactions among organs in vivo. The purpose of the in vitro experiments in this study is to provide evidence to support the clinical findings.

## Conclusions

This study demonstrated that zonulin, LPS, and LBP are practical tools for predicting AKI and HRS–AKI in patients with cirrhosis. In the in vitro experiments, zonulin, LPS, and LBP mediated apoptosis and necrosis of intestinal cells as well as the EMT and migration of renal tubular cells. Future studies should focus on the markers involved in gut-kidney crosstalk, which may be possible targets for renal outcomes in cirrhotic patients.

### Supplementary Information

Supplementary Tables.

## Data Availability

The datasets are available from the corresponding author based on reasonable request.
