# Supplementary material for: Prediction models combining zonulin, LPS, and LBP predict acute kidney injury and hepatorenal syndrome–acute kidney injury in cirrhotic patients
Source: Sci Rep. 2023 Aug 11;13:13048. doi: 10.1038/s41598-023-40088-7 (PMC10421946; doi:10.1038/s41598-023-40088-7)
Supplement: Supplementary file 1 — Supplementary Tables. [file 41598_2023_40088_MOESM1_ESM.docx]

Supplementary Table S1 Definition of clinical events

| Clinical events | Guideline | Definition |
| --- | --- | --- |
| Sepsis | Sepsis 3.0 definitions^30^ | An increase in the Sequential Organ Failure Assessment (SOFA) score of two points or more occurred after infection. |
| Septic shock | Sepsis 3.0 definitions^30^ | When vasopressor was required to maintain a mean arterial pressure of 65 mmHg or greater, and the serum lactate level was <2 mmol/L in the absence of hypovolemia. |
| Acute on chronic liver failure | Asia-Pacific Association for the study of Liver (APASL) definition^31^ | An acute hepatic insult manifesting as jaundice (serum bilirubin ≥ 5 mg/dL [85 μmol/L]) and coagulopathy (international normalized ratio (INR) ≥ 1.5 or prothrombin activity < 40%) complicated within 4 weeks by clinical ascites and/or hepatic encephalopathy in a patient with previously diagnosed or undiagnosed chronic liver disease/cirrhosis. |

| Supplementary Table S1 Primers of various genes | | |
| --- | --- | --- |
| Gene name | Forwards | Reverse |
| E-cadherin | 5′- CAGGTCTCCTCATGGCTTTGC -3′ | 5′-CTTCCGAAAAGAAGGCTGTCC-3′ |
| α-SMA | 5′-CGGGCTTTGCTGGTGATG-3′ | 5′-CCCTCGATGGATGGGAAA -3′ |
| Vimentin | 5′-AGAACCTGCAGGAGGCAGAAGAAT -3′ | 5′-TTCCATTTCACGCATCTGGCGTT-3 |
| ZO-1 | 5′- CGGGACTGTTGGTATTGGCTAGA -3′ | 5′- GGCCAGGGCCATAGTAAAGTTTG -3′ |
| Occludin | 5′- TCCTATAAATCCACGCCGGTTC -3′ | 5′- CTCAAAGTTACCACCGCTGCTG -3′ |
| Claudin-1 | 5′- GCACATACCTTCATGTGGCTCAG -3′ | 5′- TGGAACAGAGCACAAACATGTCA -3′ |
| 18S | 5′-GTAACCCGTTGAACCCCATT-3′ | 5′-CCATCCAATCGGTAGTAGCG-3 |
